# A randomized, double-blind, placebo-controlled study of B-cell lymphoma 2 homology 3 mimetic gossypol combined with docetaxel and cisplatin for advanced non-small cell lung cancer with high expression of apurinic/apyrimidinic endonuclease 1

**DOI:** 10.1007/s10637-020-00927-0

**Published:** 2020-06-11

**Authors:** Yuxiao Wang, Xuemei Li, Liang Zhang, Mengxia Li, Nan Dai, Hao Luo, Jinlu Shan, Xueqin Yang, Mingfang Xu, Yan Feng, Chengxiong Xu, Chengyuan Qian, Dong Wang

**Affiliations:** Cancer Center, Daping Hospital & Army Medical Center of PLA, Army Medical University, 400042 Chongqing, China

**Keywords:** Gossypol, Non-small cell lung cancer, APE1, Docetaxel, Cisplatin

## Abstract

*Background* Overexpression of apurinic/apyrimidinic endonuclease 1 (APE1) is an important cause of poor chemotherapeutic efficacy in advanced non-small cell lung cancer (NSCLC) patients. Gossypol, a new inhibitor of APE1, in combination with docetaxel and cisplatin is believed to improve the efficacy of chemotherapy for advanced NSCLC with high APE1 expression. *Methods* Sixty-two patients were randomly assigned to two groups. Thirty-one patients in the experimental group received 75 mg/m^2^ docetaxel and 75 mg/m^2^ cisplatin on day 1 with gossypol administered at 20 mg once daily on days 1 to 14 every 21 days. The control group received placebo with the same docetaxel and cisplatin regimen. The primary endpoint was progression-free survival (PFS); secondary endpoints included overall survival (OS), response rate, and toxicity. *Results* There were no significant differences in PFS and OS between the experimental group and the control group. The median PFS (mPFS) in the experimental and control groups was 7.43 and 4.9 months, respectively (HR = 0.54; *p* = 0.06), and the median OS (mOS) was 18.37 and 14.7 months, respectively (HR = 0.68; *p* = 0.27). No significant differences in response rate and serious adverse events were found between the groups. *Conclusion* The experimental group had a better mPFS and mOS than did the control group, though no significant difference was observed. Because the regimen of gossypol combined with docetaxel and cisplatin was well tolerated, future studies with larger sample sizes should be performed.

## Introduction

Non-small cell lung cancer (NSCLC) is the most common type of lung cancer, and more than 70% of NSCLC patients are diagnosed with advanced-stage disease (stage III, local progression or stage IV, distant metastasis); thus, the majority of these patients have lost the opportunity for surgical resection [1]. Platinum-based chemotherapy is the cornerstone of treatment for advanced NSCLC [2]. However, chemotherapy is only beneficial for a few patients with advanced NSCLC: the median progression-free survival (mPFS) is 4.2 to 5.5 months [2–4], the median overall survival (mOS) is only 8.5 to 10.5 months [4–6], and the 5-year survival rate is still less than 10% [7]. Overall, platinum-containing chemotherapeutic drugs may have reached a plateau for the treatment of advanced NSCLC, and platinum resistance may be a key factor in the limited efficacy.

The main antitumor mechanism of platinum drugs involves platinum binding to DNA to form a platinum-DNA adduct, which disrupts DNA replication via intrastrand crosslinking, resulting in DNA damage and cell death [8]. Alterations in DNA repair capacity are an important molecular basis of platinum resistance [9, 10]. The stronger is the DNA repair capacity of the tumor, the more resistant it is to chemotherapeutic drugs. Apurinic/apyrimidinic endonuclease 1 (APE1) is a biomacromolecule functional complex that has dual functions in DNA damage repair and redox [11–14]. On the one hand, APE1 is the main rate-limiting enzyme in the base excision repair pathway and is involved in the DNA damage repair of purine/pyrimidine deletion sites (AP sites) caused by oxidants and alkylating agents. On the other hand, APE1 is an important mediator in the cellular oxidative stress response pathway. By maintaining the reductive state of DNA binding region-specific cysteines and maintaining the activation and reduction of various transcription factors (such as AP-1, NF-kappa B, Myb, HIF-1α, HLF, PAX, p53), this complex is indirectly involved in the regulation of gene expression [15, 16]. Some of these transcription factors are closely related to chemotherapeutic resistance [17, 18].

Many studies have shown that APE1 expression is increased in various malignant tumors, including NSCLC [19], colorectal cancer [20], ovarian cancer [21, 22], glioma [23], cervical cancer [24], prostate cancer [25] and pancreatic cancer [26]. This increase in APE1 expression is associated with chemotherapeutic resistance [27]. Our previous study confirmed that downregulating APE1 protein expression can increase the sensitivity of A549 lung cancer cell lines to platinum [28]. Clinical data have also confirmed that the survival time of patients with low expression of APE1 is longer than that of patients with high expression of APE1 after platinum adjuvant chemotherapy [28]. Thus, inhibition of APE1 may improve the sensitivity of NSCLC to platinum-containing regimens.

Small molecule inhibitors of APE1 have become a focus of research in this field, but the APE1 inhibitors available only target one of its functions. For example, E3330 only inhibits the redox activity of APE1 but does not affect its endonuclease activity [29]; CRT0044876 only blocks its repair function [30]. In our previous screening of small molecule inhibitors of APE1, we found that gossypol might constitute a new type of inhibitor that simultaneously interferes with both functions of APE1 [31]. Therefore, gossypol combined with platinum has important clinical value in the treatment of NSCLC patients with high APE1 expression. This study is a prospective, randomized clinical trial to assess whether gossypol can enhance the efficacy of docetaxel and cisplatin in patients with high expression of APE1, to provide a new target for targeted chemotherapy of NSCLC and to test a safe and economical combination of drugs to improve the efficacy of advanced NSCLC chemotherapy.

## Materials and methods

### Diagnostic criteria

The diagnosis was made according to the 6th Edition of Oncology Diagnostics and the 6th Edition of Internal Medicine published by the People’s Health Publishing House of the People’s Republic of China.

### Inclusion criteria

Eligible patients were confirmed to have advanced NSCLC (IIIB/IV) with evaluable lesions by histology or cytology. High expression of APE1 (+++) was detected by immunohistochemistry. All patients, aged between 18 and 75 years, had not previously received platinum-based chemotherapy and had no epidermal growth factor receptor (EGFR) mutations or anaplastic lymphoma kinase (ALK) positivity. The patients may have had a history of brain/meningeal metastasis but underwent local treatment (surgery/radiotherapy) before randomization and were clinically stable for at least 2 months. Eligibility criteria included a performance status (PS) of 0 to 1 and an estimated survival time ≥ 3 months. Routine blood examination results were as follows: hemoglobin ≥ 90 g/L (within 14 days without blood transfusion), absolute neutrophil count ≥ 1.5 × 10^9^/L, and platelet ≥ 80 × 10^9^/L. Biochemical analyses met the following criteria: (1) bilirubin < 1.25 × the upper limit of normal (ULN); (2) alanine aminotransferase and aspartate transaminase < 2.5 × ULN (with liver metastasis, alanine aminotransferase and aspartate transaminase < 5 × ULN); and (3) serum creatinine ≤ 1.25 ml/min; creatinine clearance > 45 ml/min. This trial was consistent with the Helsinki Declaration and was approved by the Ethics Committee of Daping Hospital & Army Medical Center of PLA.

### Drug source

The experimental drug was compound gossypol acetate tablets (specification: 20 mg/tablet), which were produced by Xi’an Northern Pharmaceutical Co., Ltd. The control drug was a placebo (gossypol simulated tablet), which was developed and provided by Jiangsu Yasheng Pharmaceutical Development Co., Ltd. The specifications of docetaxel, which was produced by Jiangsu Hengrui Pharmaceutical Co., Ltd., were 20 mg/bottle. Cisplatin was produced by Jiangsu Hausen Pharmaceutical Co., Ltd. with specifications of 10 mg/bottle.

### Treatment

The study was randomized and placebo controlled (clinical trial information: NCT01977209), and the patients were randomly assigned to two groups. The experimental group received 75 mg/m^2^ docetaxel and 75 mg/m^2^ cisplatin on day 1 with 20 mg gossypol once daily from days 1 to 14 every 21 days. The control group received the same docetaxel and cisplatin regimen as the placebo. Corticosteroids and 5-hydroxytryptamine receptor antagonists were administered on the first and second days of chemotherapy. Patients received at least 4 cycles of treatment in the event of unacceptable toxicity or progressive disease (PD). The full analysis set (FAS) included all patients who had been treated with at least one cycle of treatment and left imaging data records. Effectiveness was evaluated every 6 weeks by computed tomography or other imaging modalities. If the subjects discontinued treatment before the appearance of PD, they were followed up every 42 days for imaging examination to determine if the tumor was progressing. No other antitumor therapy was administered before PD.

### Response and toxicity evaluation

NCI-CTC2.0 (National Cancer Institute Common Toxicity Criteria version 2.0) and RECIST1.1 (Response Evaluation Criteria in Solid Tumors guidelines version 1.1) were used to evaluate and grade toxicity and to assess response, respectively. All imaging data were retained and submitted to the independent imaging evaluation committee, which was not related to the trial, to evaluate the curative effect.

### Study endpoint and follow-up

Progression-free survival (PFS) was the primary endpoint of the study. The secondary endpoints were overall survival (OS), objective response rate (ORR), disease control rate (DCR), toxicity and safety. PFS was defined as the time between the date on which the patient was randomly enrolled into a group and the date of any recorded PD or death from any cause. Recurrence, appearance of new lesions or death was considered the end of the study for that case, and the use of other systemic or target antitumor therapies was also considered PD. For patients who had not yet experienced PD or death at the end of the study, the time of the last record was considered to be censored data. For patients who were lost to follow-up without PD, these data were also censored if the imaging data were recorded.

### Statistical analysis

The sample size was calculated under the assumption that mPFS increased from 4.5 months to 8 months. With an α error of 5%, a β error of 20%, and a predicted 10% of cases lost to follow-up, our anticipated sample size was 256 cases.

All statistical analyses were performed with Statistical Package for Social Sciences (SPSS, Version 23) software. Intent-to-treat (ITT) analysis was performed for data for all patients who entered the study and received treatment. T-tests, chi-square tests and Fisher’s exact tests were employed to evaluate whether the baseline characteristics of the two groups were comparable. OS and PFS were evaluated by stratified log-rank tests, and the results are summarized by a Kaplan-Meier survival curve. All statistical tests were two-sided, and a p value ≤ 0.05 was considered significant. Furthermore, we calculated the hazard ratio (HR) with the 95% confidence interval (CI).

## Results

### Patient characteristics

**Fig. 1:**
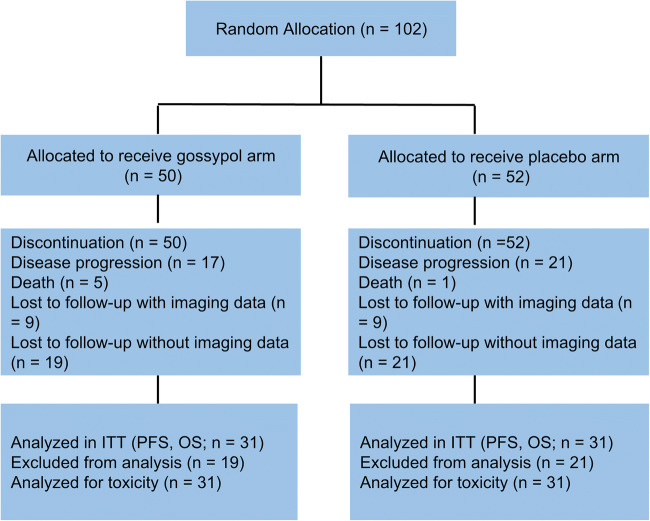
Ensemble diagram. ITT, intent-to-treat; PFS, progression-free survival; OS, overall survival

From January 2014 to February 2017, only 102 patients signed informed consent and participated in the study (50 in the experimental group and 52 in the control group). However, because of the poor compliance of patients with advanced NSCLC, 40 were lost to follow-up after only one chemotherapy session without reexamination (19 in the experimental group and 21 in the control group); as no imaging data were available, these cases were excluded. Only 62 cases were in accordance with the analysis. Sixty-two patients (ITT set) were randomly assigned to two groups: 31 received gossypol plus docetaxel and cisplatin, and 31 received the placebo plus docetaxel and cisplatin; all patients received at least one cycle of treatment (Fig. 1). In the placebo group, 31 patients received a total of 88 cycles, with a mean of 2.8 cycles. In the experimental group, fifteen patients (48%) completed four cycles of gossypol plus docetaxel and cisplatin, 8 patients (26%) received three cycles, seven (23%) received two cycles, and 1 (3%) received one cycle.

Table 1 lists the baseline characteristics of the patients, and the two groups of the study were demographically balanced, with no significant differences (*p* > 0.05). Among all patients, 38 had stage IIIb disease (20 adenocarcinoma, 17 squamous, 1 sarcomatous carcinoma), and 24 had stage IV disease (21 adenocarcinoma and 3 squamous). In the gossypol group, there were 23 males, with a mean age of 56.25 years. Among the 31 patients, 18 smoked, 25 had a PS score of 1, and 5 had only 1 metastatic disease site. The placebo group included 6 females, with a mean age of 59.41 years; 12 were nonsmokers, 7 had a PS score of 0, and 19 had at least 2 metastatic disease sites.

**Table 1:** Demographic data for enrolled patients

	Gossypol + Docetaxel and Cisplatin	Placebo+ Docetaxel and Cisplatin	P
	N = 31	N = 31	
Age			0.190
Mean ± SD	56.25 ± 9.70	59.41 ± 9.07	
Gender, n (%)			0.544
Male	23(74)	25(81)	
Female	8(26)	6(19)	
Smoking status, n (%)			0.796
Non-smoker	13(42)	12(39)	
Smoker	18(58)	19(61)	
ECOG PS, n (%)			0.755
0	6(19)	7(23)	
1	25(81)	24(77)	
NSCLC Stage, n (%)			1.000
IIIb	19(61)	19(61)	
IV	12(39)	12(39)	
Histology, n (%)			1.000
Squamous	10(32)	10(32)	
Adenocarcinoma	20(65)	21(68)	
Sarcomatoid carcinoma	1(3)	0	
No. of metastatic disease sites, n (%)			0.581
0	5(16)	3(10)	
1	7(23)	10(32)	
≥ 2	19(61)	18(58)	

### Comparison of short-term efficacy between the two groups

All 62 patients were evaluated for response. No complete responses (CRs) were observed in this study. There were 3 and 1 cases of partial response (PR) in the gossypol and placebo groups, respectively. This finding corresponds to ORRs of 9.7% and 3.2%, respectively. The best response recorded as SD was observed in 23 and 18 patients in the gossypol and placebo groups, respectively. The DCRs were 83.9% and 61.3%, respectively (Table 2).

**Table 2:** Summary of response rate

	Gossypol + Docetaxel and Cisplatin	Placebo+ Docetaxel and Cisplatin
	(N = 31)	(N = 31)
CR	0	0
PR	3	1
SD	23	18
PD	5	12
ORR	9.7%	3.2%
DCR	83.9%	61.3%

CR, complete response; PR, partial response; SD, stable disease; PD, progressive disease; ORR, objective response rate; DCR, disease control rate

### Comparison of long-term efficacy between the two groups

**Fig. 2:**
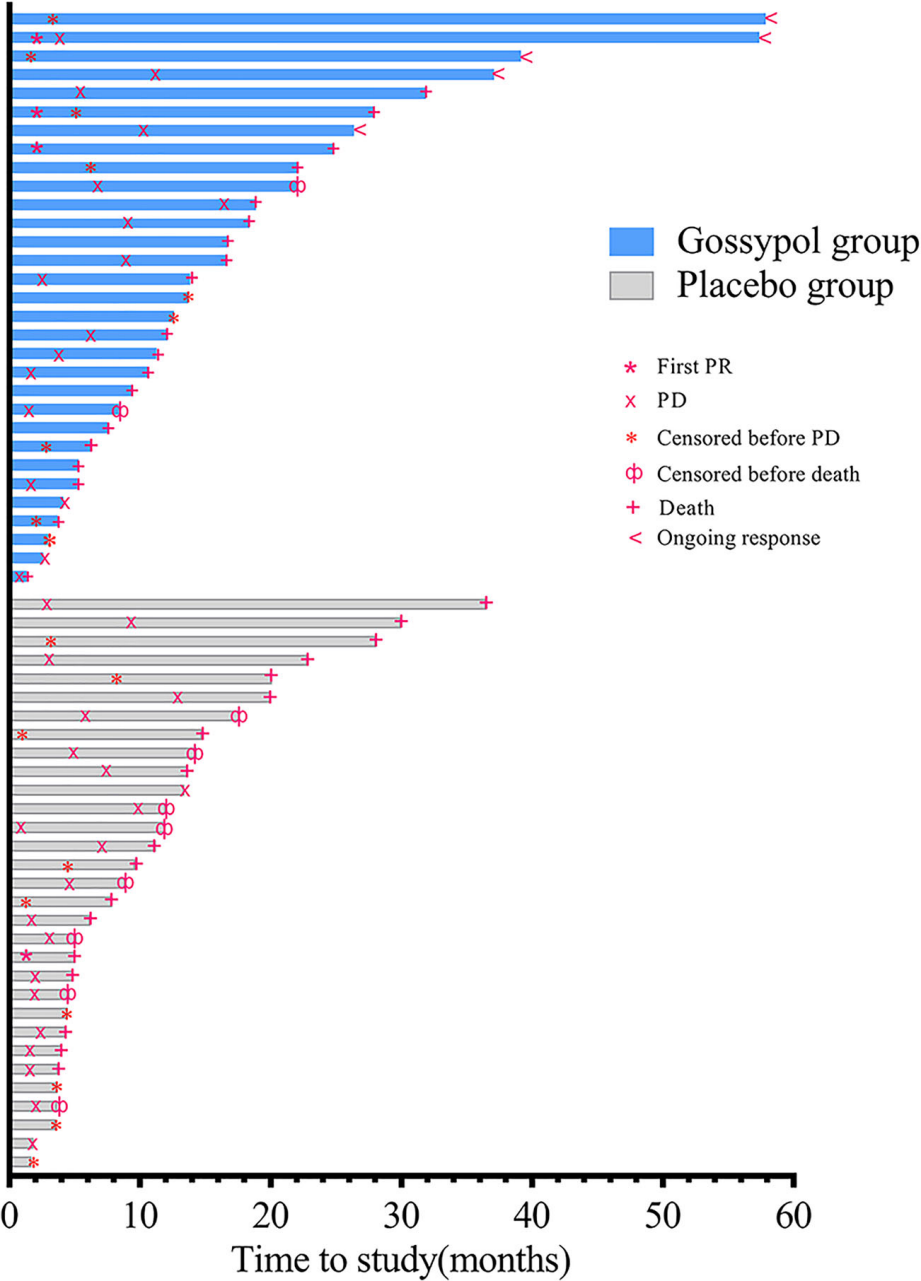
The therapeutic process and outcomes for evaluable patients

Forty-four patients (72%), 22 in the gossypol group and 22 in the placebo group, had an event for PFS. In the gossypol group, 17 patients had objective PD, five died due to progression, and 9 were lost to follow-up before PD. In the placebo group, 21 patients had objective PD, one died without objective PD, and nine were lost to follow-up. For OS, 36 patients died, and 26 patients were censored (5 patients with ongoing response). Figure 2 shows the therapeutic process and outcomes for the evaluable patients.

**Fig. 3:**
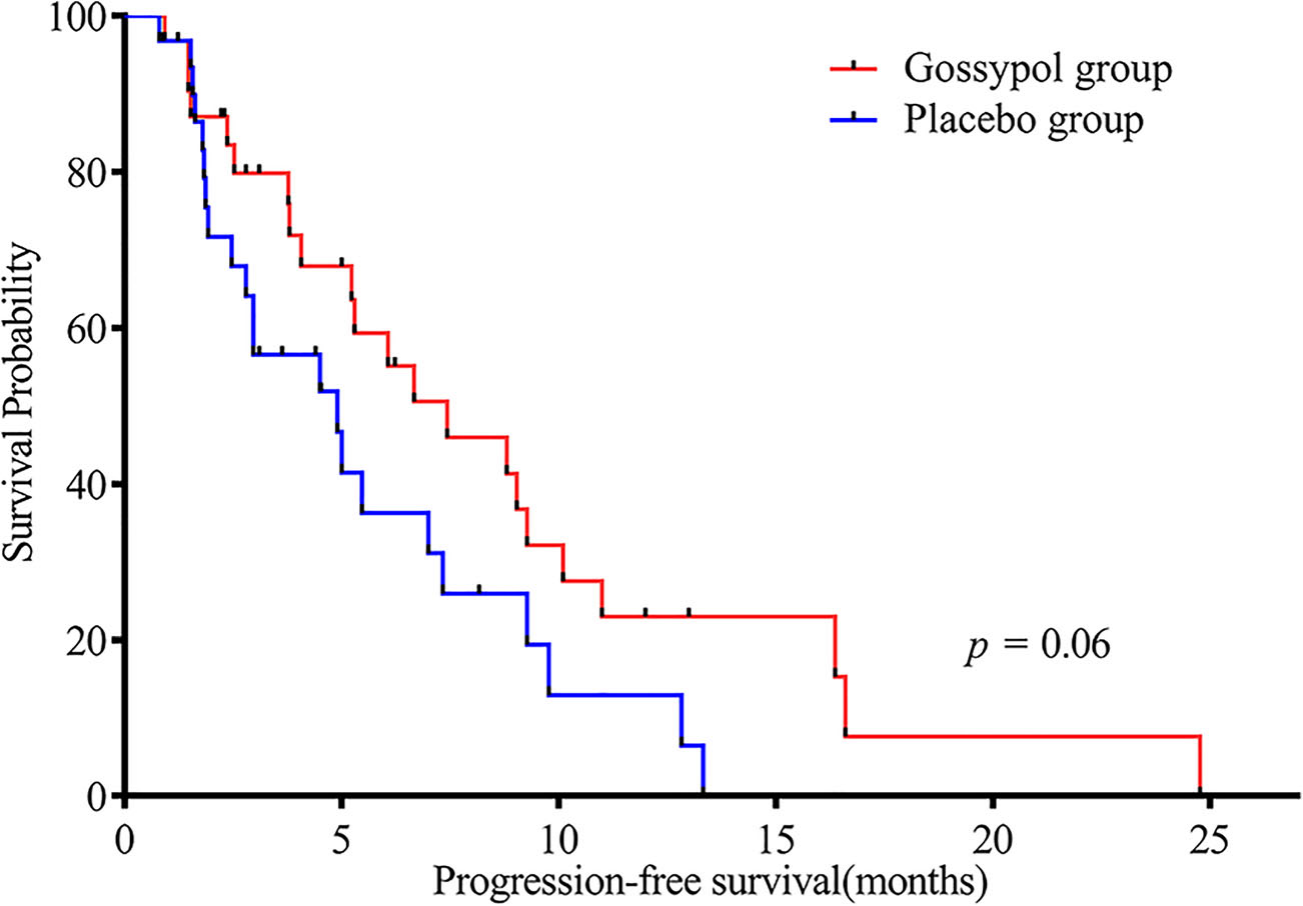
Kaplan-Meier estimates of progression-free survival

**Fig. 4:**
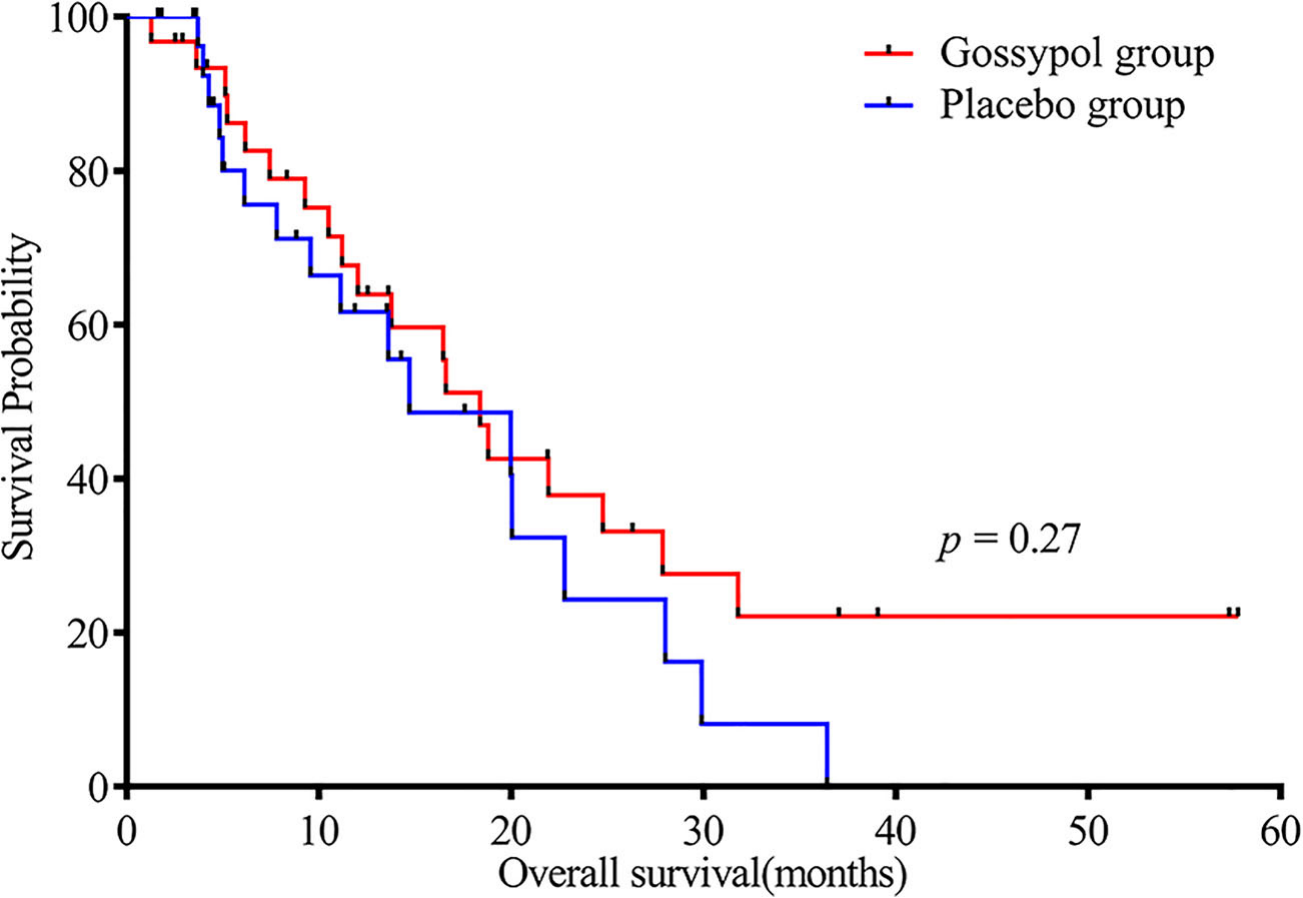
Kaplan-Meier estimates of overall survival

mPFS in the gossypol and placebo groups was 7.43 and 4.9 months, respectively [*p* = 0.06, HR (95% CI): 0.54 (0.29–1.03)]. The 6-month PFS rate in the gossypol group was 45.2% (Fig. 3), which was higher than that in the placebo group (22.6%). mOS in the gossypol and placebo groups was 18.37 and 14.7 months, respectively [*p* = 0.27, HR (95% CI): 0.68 (0.34–1.34)]. The benefits for OS remained unchanged during longer follow-up. There were 17 vs. 10 patients who survived more than 12 months in the gossypol group vs. the placebo group (Fig. 4).

### Safety and toxicity

There were no treatment-related deaths or discontinuation of treatment due to toxicity. As shown in Table 3, only 1 patient had grade 3 anemia in the gossypol group, but we did not reduce the dose level because the hemoglobin level in that patient later returned to normal. Most patients had mild events (grade 1 or 2 toxicity): the number of patients with anemia in the placebo group was greater than that in the gossypol group (7 vs. 3); 2 patients had neutropenia and three had asthenia in the gossypol group, and 3 patients had leukopenia and three patients had thrombocytopenia in the placebo group. There was no significant increase in toxicity in the gossypol group compared with the placebo group.

**Table 3:** The occurrence of adverse events

Adverse	Gossypol + Docetaxel and Cisplatin		Placebo + Docetaxel and Cisplatin	
Events	Grade I-II, N (%)	Grade III-IV, N (%)	Grade I-II, N (%)	Grade III-IV, N (%)
Fatigue	4(12.9)	0	5(16.1)	0
Asthenia	3(9.7)	0	2(6.5)	0
Dyspnea	2(6.5)	0	3(9.7)	0
Anemia	3(9.7)	1(3.2)	7(22.6)	0
Neutropenia	2(6.5)	0	0	0
Leukopenia	4(12.9)	0	3(9.7)	0
Thrombocytopenia	3(9.7)	0	3(9.7)	0
Headache	2(6.5)	0	2(6.5)	0

## Discussion

This study was intended to improve the primary endpoint of PFS. However, the results showed no significant differences in PFS and OS between the gossypol and placebo groups (*p >* 0.05). Nonetheless, the DCR for the gossypol group improved by 22.6% compared with that of the placebo group. Moreover, gossypol combined with docetaxel and cisplatin was well tolerated, and there was no significant increase in toxicity for the gossypol group compared with the placebo group. The vast majority of events for this study were grade 1 or 2 toxicity, and only one patient with grade 3 neutropenia was found in the experimental group.

Unlike targeted drugs for the treatment of NSCLC, chemotherapeutic drugs (including platinum) are usually administered based on the response rate in previous trials to cancer patients who do not have any specific gene targets. A comparison of serum APE1 protein levels of 523 healthy blood donors with APE1 protein levels in biopsies from 172 NSCLC patients and serum of 412 NSCLC patients receiving platinum chemotherapy showed that APE1 is a predictive biomarker for NSCLC prognosis and treatment effect [32]. Another clinical study was conducted to confirm the role of APE1 in the chemosensitivity of NSCLC to the platinum regimen [33], with 172 patients with advanced NSCLC receiving at least two cycles. APE1 protein expression was detected by immunohistochemistry and used to evaluate the relationship between protein expression and platinum chemotherapy. The results showed an APE1 positivity rate of 75.74%. The chemotherapeutic response rate of patients without expression of APE1 was 48.48%, which was significantly higher than that of APE1-positive patients (26.21%). The mPFS of patients without APE1 expression was significantly longer than that in APE1-positive patients (11.1 vs. 8.4 months and *p* = 0.008). Gossypol, an inhibitor of APE1, is a natural compound extracted from cottonseed that was originally used as an antifertility agent and later as a cytotoxic agent [34–36]. It has been proven to be a antineoplastic drug that can act on various cancers [37–40]. Although the antitumor mechanism of gossypol has not been fully clarified, its molecular targets are gradually being revealed. As an inhibitor of various B-cell lymphoma 2 (Bcl-2) molecules and a nonselective Bcl-2 homology 3 (BH3) analog, gossypol can bind to the BH3 domain of members of the Bcl-2 family (such as Bcl-2, Bcl-XL, Mcl-1, Bcl-W), inactivate their functions and promote apoptosis [41]. Gossypol not only specifically binds to intracellular BH3 sites and replaces apoptotic proteins such as Bax/Bak, which can indirectly inhibit the activities of Bcl-2 and Bcl-XL, but it also directly binds to the BH3 sites of proapoptotic proteins such as Bax and Bak to promote apoptosis [42]. Importantly, some studies have found that Bcl-2 directly interacts with APE1 through its BH domain [43], which might be the mechanism by which gossypol acts as an APE1 inhibitor. In our previous study [31], we also showed by dual polarization interferometry technology that gossypol was able to interact directly with APE1 and inhibit the repair activity and redox function of APE1. Furthermore, in human cancer cell lines, gossypol was more effective at killing cancer cells with APE1 overexpression than negative controls, and the combination of gossypol and cisplatin resulted in enhanced cell killing and higher antitumor activity in vivo than cisplatin alone. All of these findings might explain why the gossypol group was superior to the control group with regard to both PFS and OS, and we believe that gossypol combined with the platinum regimen has important clinical value.

In this study, the mPFS of the gossypol group was 2.53 months longer than that of the placebo group (7.43 vs. 4.9 months), and the mOS of the gossypol group increased by 3.67 months compared with that of the placebo group (18.37 vs. 14.7 months). Although these parameters of the gossypol group were better than those of the placebo group, there were no significant differences between the two groups for the following reasons. First, the sample size of this experiment was small. Many patients with advanced NSCLC were treated with chemotherapy after diagnosis in the respiratory department or thoracic surgery, and there were few patients who had not received a first-line platinum regimen directly from the oncology department. Second, many censored cases were included. A total of 18 patients were lost to follow-up before PD, which led to no significant difference between the groups. Similarly, although the DCR in the gossypol group was significantly higher than that in the placebo group, there were no CRs and few cases of PR, which might have been due to the large number of censored cases resulting in insufficient treatment cycle and no corresponding efficacy observed.

Gossypol has been shown to have good safety and tolerance profiles [44, 45]. For instance, clinical trials indicate that gossypol is apparently safe at a dosage of 40 mg/day [37]. A lower dose of 20 mg once daily was chosen in the present study. Compared with previous studies on gossypol [46], our results showed no increase in gastrointestinal toxicities, cardiac rhythm abnormalities, liver damage, or other severe adverse events. Because the compound gossypol acetate tablets provided to the experimental group contain potassium chloride, the patients did not show hypokalemia, which is the most common side effect of gossypol [47]. Only one patient in the gossypol group had grade 3 anemia and hemoglobin of 78 g/L, which may have been caused by gossypol-induced suicidal erythrocyte death [48].

The drugs used in our trial (gossypol, docetaxel and cisplatin) have the advantages of being easily available and affordable. Platinum-containing chemotherapy is the basis for the treatment of advanced NSCLC, and thus, gossypol might be combined with most first-line chemotherapeutic drugs. One limitation of our study was that it was carried out in a single institution. The patients were mostly from Southwest China and were not diverse. Moreover, although all imaging was assessed by seasoned thoracic oncologic radiologists, our trial lacked a centralized, blinded radiology review.

In conclusion, gossypol, as a new small molecule inhibitor of APE1, has the advantages of abundant sources, low cost, high safety, and low toxicity in clinical applications. Although there was no significant difference, the gossypol group had better outcomes of increased mPFS and mOS than the placebo group. The lack of significant differences between the groups may be due to the sample size and the censored data. In the future, we will expand the sample size and improve the follow-up rate of patients to observe the curative effect of this treatment.

## Data Availability

The data and materials used in this study are available from the corresponding author on reasonable request.

## Abbreviations

NSCLC: Non-small cell lung cancer
PFS: Progression-free survival
OS: Overall survival
mPFS: Median progression-free survival
mOS: Median overall survival
APE1: Apurinic/apyrimidinic endonuclease 1
PS: Performance status
EGFR: Epidermal growth factor receptor
ALK: Anaplastic lymphoma kinase
ULN: Upper limit of normal
PD: Progressive disease
FAS: Full analysis set
NCI-CTC2.0: National Cancer Institute Common Toxicity Criteria version 2.0
RECIST1.1: Response Evaluation Criteria in Solid Tumors guidelines version 1.1
ORR: Objective response rate
DCR: Disease control rate
ITT: Intent-to-treat
HR: Hazard ratio
CR: Complete response
PR: Partial response
SD: Stable disease
CI: Confidence interval
Bcl-2: B-cell lymphoma 2
BH3: Bcl-2 homology 3
